# Intestinal epithelial dysplasia (tufting enteropathy)

**DOI:** 10.1186/1750-1172-2-20

**Published:** 2007-04-20

**Authors:** Olivier Goulet, Julie Salomon, Frank Ruemmele, Natacha Patey-Mariaud de Serres, Nicole Brousse

**Affiliations:** 1 Department of Pediatric Gastroenterology-Hepatology and Nutrition and Reference Center for Rare Digestive Disease, Hopital Necker-Enfants Malades, 149, Rue de Sèvres, Cédex 15, 75743 Paris, France; 2 Department of Pathology, Hopital Necker-Enfants Malades, 149, Rue de Sèvres, Cédex 15, 75743 Paris, France

## Abstract

Intestinal epithelial dysplasia (IED), also known as tufting enteropathy, is a congenital enteropathy presenting with early-onset severe intractable diarrhea causing sometimes irreversible intestinal failure. To date, no epidemiological data are available, however, the prevalence can be estimated at around 1/50,000–100,000 live births in Western Europe. The prevalence seems higher in areas with high degree of consanguinity and in patients of Arabic origin. Infants develop within the first days after birth a watery diarrhea persistent in spite of bowel rest and parenteral nutrition. Some infants are reported to have associated choanal rectal or esophageal atresia. IED is thought to be related to abnormal enterocytes development and/or differentiation. Nonspecific punctuated keratitis was reported in more than 60% of patients. Histology shows various degree of villous atrophy, with low or without mononuclear cell infiltration of the lamina propria but specific histological abnormalities involving the epithelium with disorganization of surface enterocytes with focal crowding, resembling tufts. Several associated specific features were reported, including abnormal deposition of laminin and heparan sulfate proteoglycan (HSPG) in the basement membrane, increased expression of desmoglein and ultrastructural changes in the desmosomes, and abnormal distribution of **α**2**β**1 integrin adhesion molecules. One model of transgenic mice in which the gene encoding the transcription factor Elf3 is disrupted have morphologic features resembling IED. Parental consanguinity and/or affected siblings suggest an autosomal recessive transmission but the causative gene(s) have not been yet identified making prenatal diagnosis unavailable. Some infants have a milder phenotype than others but in most patients, the severity of the intestinal malabsorption even with enteral feeding make them totally dependent on daily long-term parenteral nutrition with a subsequent risk of complications. IED becomes an indication for intestinal transplantation, while timing of referral for it is crucial before the onset of severe complications.

## Disease name and synonyms

Intestinal epithelial dysplasia

Tufting enteropathy

Congenital enteropathy

Congenital familial intractable diarrhea with enterocytes assembly abnormalities

## Definition

Intestinal epithelial dysplasia (IED), also known as tufting enteropathy, is a congenital enteropathy presenting with early-onset severe intractable diarrhea and persistent villous atrophy with low or without mononuclear cell infiltration of the lamina propria but specific histological abnormalities involving the epithelium [1,2]. IED is characterized by clinical and histological heterogeneity and association with malformations or other epithelial diseases. It is thought to be related to abnormal enterocytes development and/or differentiation.

## Epidemiology

IED appears to be more common than microvillous atrophy (MVA), also known as microvillous inclusion disease (MVID), especially in Middle-East, however, it remains very rare. Many cases are not yet recognized since the description of this disorder is recent. To date, no epidemiological data are available, however, the prevalence can be estimated at around 1/50,000–100,000 live births in Western Europe. The largest cohort of patients is currently being treated at the Necker-Enfants Malades Hospital in Paris, France. The prevalence is higher in countries with high degree of consanguinity. Our studies indicate that IED is frequent in patients of Arabic origin (Middle-East, Turkey and North Africa). The prevalence is also high in the island of Malta (in the Mediterranean sea) but the phenotype appears to be milder.

## History of the description

IDE is a newly described clinicopathologic entity with intractable diarrhea in infants. Three cases of neonatal severe diarrhea with abnormal epithelial pictures were first reported by Reifen *et al*., 1994, under the name of 'tufting enteropathy" [3]. We identified nine cases of severe neonatal diarrhea, which were clearly distinguishable from MVA and resembled "tufting enteropathy" [4]. Further studies of these patients allowed us to identify IED as a constitutive epithelial disorder involving both small intestine and colon [5]. A main characteristic of this disease is its clinical and histological heterogeneity and its association with malformations or other epithelial diseases.

## Clinical description, associated disorders and diagnostic criteria

In general, infants with IDE develop watery diarrhea within the first days after birth. It is severe in most of the cases. Stool volumes may be as high as 100–200 ml/kg body weight per day, with electrolyte concentrations similar to those seen in small intestinal fluid. In rare cases the diarrhea may be less abundant and sometimes may mislead the diagnosis [5]. The growth is impaired. There is no past history of hydramnios suggesting congenital chloride diarrhea or sodium malabsorption diarrhea. Most patients have consanguineous parents and/or affected siblings, some of whom died during the first months of life from severe diarrhea of unknown origin.

Several cases of IED have been reported as being associated with phenotypic abnormalities, for example Dubowitz syndrome or malformative syndrome [6-8]. Some affected children are reported to have dysmorphic facial features [6]. An association between congenital intractable diarrhea of infancy (IDI) and choanal atresia has been reported in four children [7]. We have observed malformations, including esophageal atresia, choanal atresia, or unperforated anus. Moreover, nonspecific punctuated keratitis is observed in more than 60% of patients [8]. This associated keratitis is very intriguing since it is also an epithelial disease and therefore studies of keratitis might help to elucidate the molecular mechanisms of the intestinal epithelial disease. The fact that some children have ophthalmological symptoms and keratitis highlights the heterogeneity of IED (Salomon *et al*., manuscript in preparation).

## Histological presentation

Histological abnormalities in IED include villous atrophy, disorganization of the surface epithelium and basement membrane abnormalities.

### Villous atrophy

Villous atrophy is present in all patients but is variable in severity. Repeated biopsies are required.

### Epithelium

**Figure 1:**
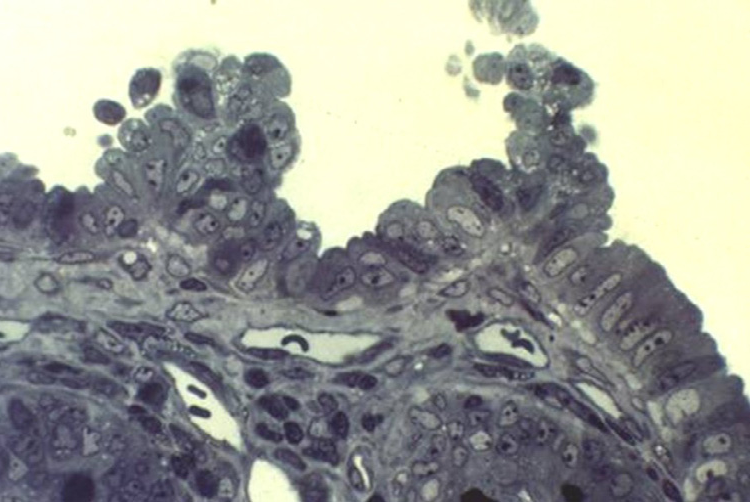
Typical disorganization of surface enterocytes with focal crowding forming tuft.

In the typical form, abnormalities are localized mainly in the epithelium and include disorganization of surface enterocytes with focal crowding, resembling tufts (Figure 1). These characteristic "tufts" of extruding epithelium, first described by Reifen *et al*. [3], are seen towards the villous tip and may affect up to 70% of villi.

### Colonic disease

Tufts are not limited to the small intestine and also involve the colonic mucosa [4].

### Specific features

**Figure 2:**
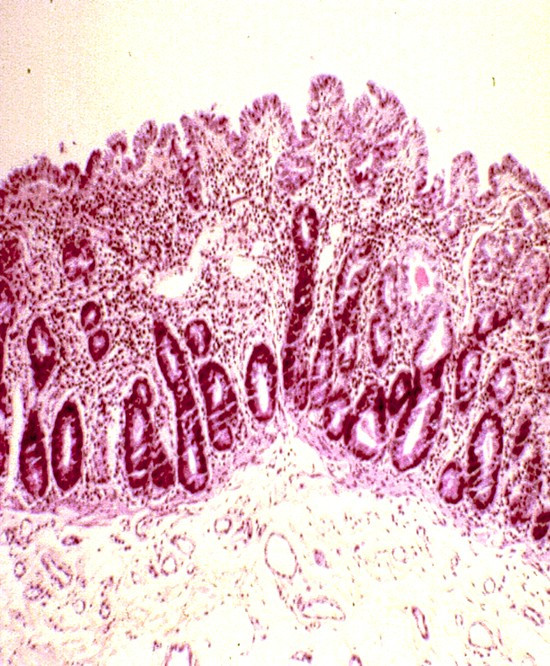
Intestinal epithelial dysplasia. Partial villous atrophy with crypt hyperplasia and/or pseudocystic crypt appearance, branching pictures and disorganization of surface epithelium.

**Figure 3:**
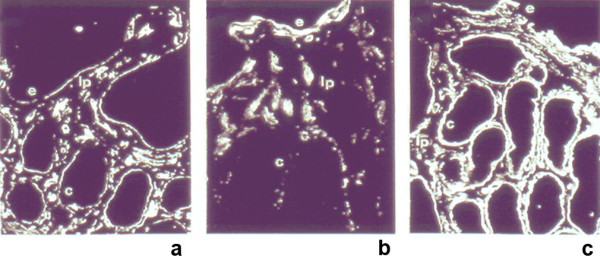
Normal deposition of laminin in control (a) but very feint and lamellar in IED (b) while heparan sulfate proteoglycan (HSPG) is overexpressed in the basement membrane.

**Figure 4:**
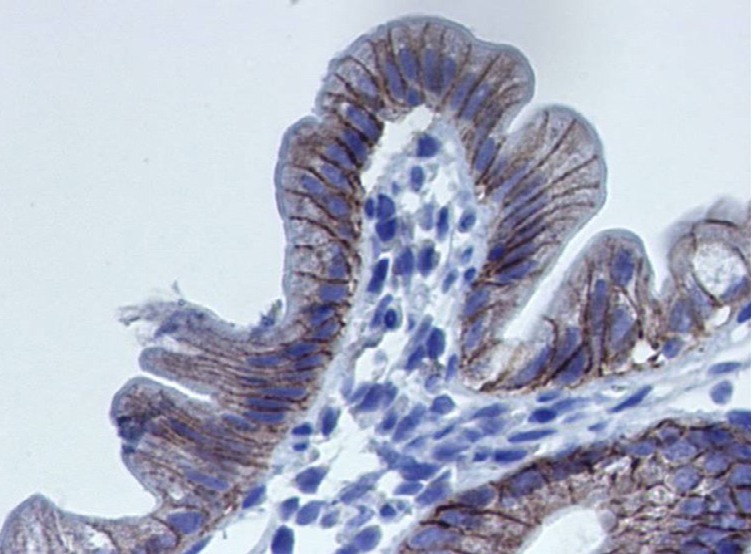
Intestinal epithelial dysplasia. Increased expression of desmoglein staining of the tight junction in a patient with intestinal epithelial dysplasia.

**Figure 5:**
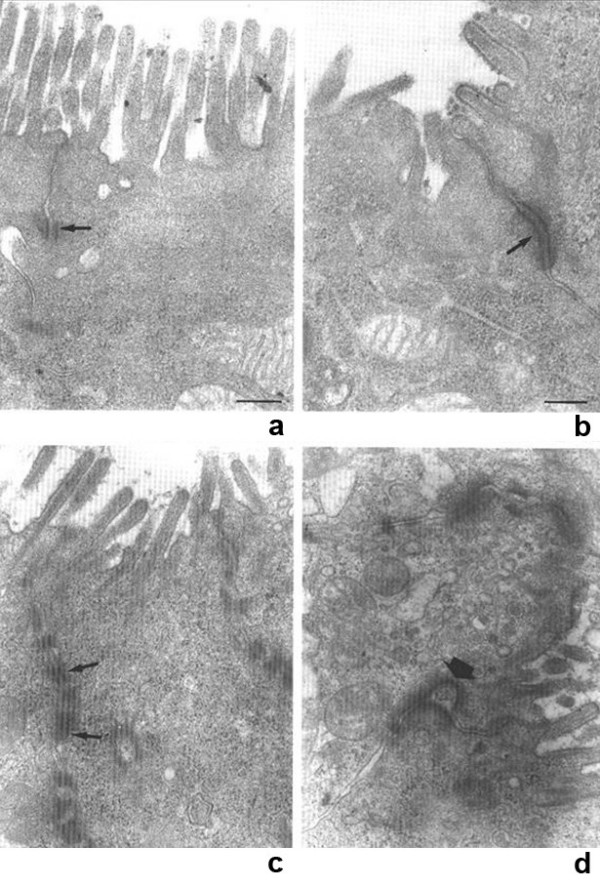
Different pictures showing ultrastructural changes in the desmosomes, which are increased in length and number.

Focal enterocyte crowding can also be observed in the crypt epithelium and, in addition, crypts often have an abnormal aspect: they are dilated with features of pseudo cysts and abnormal regeneration with branching (Figure 2) [4]. A study of biopsy specimens demonstrated that the deposition of laminin and heparan sulfate proteoglycan (HSPG) in the basement membrane was abnormal in patients with IED, compared to that from patients with celiac disease or autoimmune enteropathy [4]. Relative to the controls, there was faint and irregular laminin deposition at the epithelial lamina propria interface and the HSPG appeared large and lamellar, suggesting that abnormal development of basement membrane was at the origin of the epithelial abnormalities (Figure 3a–b–c). In addition, we observed an increased immunohistochemical expression of desmoglein in IED patients (Figure 4) and ultrastructural changes in the desmosomes, which were increased in length and number [5] (Figure 5a–b–c–d).

## Differential diagnosis

Neonatal early-onset severe diarrhea may lead to suspect ions transport defects. However, congenital chloride diarrhea (CLD) or congenital sodium diarrhea (CSD) can be easily distinguished from the absence of hydramnios and by blood and stool electrolytes assessment [9-11]. CLD is a rare autosomal recessive disorder of intestinal Cl/HCO3 exchange caused by mutations in the *SLC26A3 *gene and characterized by persistent Cl rich diarrhea from birth [9,10]. Patients with CLD present with lifetime watery diarrhea with a high Cl content and low pH, causing dehydration and hypochloremic metabolic alkalosis. Chloride is low in urine and very high in stools (Chloride > 150 mmol/L > Sodium).

CSD is caused by defective sodium/proton exchange with only few sporadic cases reported [11]. The genetics of the disease have not yet been established. Patients with CSD have acidosis and hyponatremia, and stools with high concentrations of HCO3 and sodium (150 mmol/L).

Glucose-galactose malabsorption (GGM) is an autosomal recessive disease that presents in newborn infants as a life-threatening diarrhea [12]. The diarrhea ceases within 1 h of removing the oral intake of lactose, glucose, and galactose, but promptly returns with the introduction of one or more of the offending sugars into the diet.

IED should be suspected in neonates with early-onset intractable diarrhea persisting at bowel rest. According to the early onset of diarrhea, MVA can be suspected. MVA is a congenital disorder of the intestinal epithelial cells that presents with persistent life-threatening watery diarrhea and is characterized by morphological enterocyte abnormalities [13]. MVID manifests either in the first days of life (early-onset form) or in the first two months of life (late-onset form). MVID is a very rare disorder of unknown origin, probably transmitted as an autosomal recessive trait. Light microscopy shows accumulation of PAS-positive granules at the apical pole of immature enterocytes, together with atrophic band indicating microvillus atrophy and, in parallel, an intracellular PAS or CD10 positive line. Ultrastructural analyses reveal a partial to total atrophy of microvilli on mature enterocytes with apical accumulation of numerous secretory granules in immature enterocytes.

Sometimes, histopathological presentation of IED does not show evidence of tufts. Diagnosis can be made by performing repeated intestinal biopsies. Indeed, biopsies change from being near-normal in early life (showing only signs of non specific villous atrophy, with or without monocellular cell infiltration of the lamina propria) to revealing the characteristic tufts. In addition, specific abnormalities of basement membrane components (integrins or desmosomes) in parts of mucosa are rare and difficult to detect in the absence of tufts [5]. In patients with neonatal diarrhea and villous atrophy in which MVA has been ruled out, the evidence of a punctuated keratitis is very relevant for the diagnosis of IED, since more than 60% of IED have this association. IED differs from the disease reported in a newborn presenting with pyloric atresia, intractable diarrhea and severe dermatitis which was thought to be related to a congenital deficiency of **α**6**β**4 integrin [15].

Another difficulty is related to T-cell infiltration of the lamina propria. This is especially problematic when the tufts are missing and supports the hypothesis of an immune-related enteropathy. In a mouse model of dysfunctional E-cadherin, this primary disorder of epithelial permeability or integrity was responsible for secondary T-cell mediated mucosal damage [16]. Murch *et al*. described these types of lesions in infants with epithelial dysplasia [17]. Despite the lack of evidence from clinical studies, increased intestinal permeability with subsequent antigen epithelial crossing might explain immune reaction within the lamina propria. Finally, this inflammation reaction with missing or hard-to-find tufts often leads to treat the disease as an immune enteropathy. In our experience, several children have been referred with the diagnosis of autoimmune enteropathy unresponsive to immunosuppressive treatment and severe iatrogenic symptoms from long-term steroids treatment.

## Etiopathogenesis

IED has been shown to be associated with an abnormal basement membrane [3]. Basement membrane molecules are involved in epithelial mesenchymal cell interactions, which are instrumental in intestinal development and differentiation [18-23]. Alterations suggestive of abnormal cell-cell and cell-matrix interactions have been seen in patients with IED without any evidence for abnormalities in epithelial cell polarization and proliferation [4]. Alterations included abnormal distribution of **α**2**β**1 integrin adhesion molecules along the crypt-villous axis. The **α**2**β**1 integrin is involved in the interaction of epithelial cells with various basement membrane components, such as laminin and collagen. To date, the pathophysiological mechanisms resulting in the increased immunohistochemical expression of desmoglein and the ultrastructural changes of the desmosomes are unclear [4]. Mice in which the gene encoding the transcription factor Elf3 is disrupted have morphologic features resembling epithelial dysplasia in infants [24]. In this model, there is abnormal morphogenesis of the villi, while progenitor crypt cells appear normal. The enterocytes produce low levels of transforming growth factor-beta type 2 receptor, which induces the differentiation of immature intestinal epithelia. Both the clinical studies and the findings in experimental animal models should provide clues to the pathogenesis of these epithelial abnormalities and to the cause of the severity of this neonatal diarrhea. Tufts correspond to nonapoptotic epithelial cells at the villous tips that are no longer in contact with the basement membrane. It can be speculated that a defect in normal enterocyte apoptosis at the end of their lifespan or altered cell-cell interactions are responsible for this effect. The primary or secondary nature of the formation of tufts remains to be determined.

## Genetic counseling and antenatal diagnosis

The clear association between the occurrence of IDE and the presence of parental consanguinity and/or affected siblings strongly suggests a genetic origin of this disorder. These features suggest an autosomal recessive transmission. Genetic counseling may be based on the very probable autosomal recessive mode of transmission. Since the causative gene(s) have not been yet identified, prenatal diagnosis is not possible.

## Management and outcome

IDE may be life-threatening, since the massive diarrhea leads to rapid dehydration and electrolyte imbalance, with subsequent metabolic decompensation within a few days after birth. Diarrhea persists at a lower level despite bowel rest. Attempts at continuous enteral feeding (CEF) with a protein hydrolysate or amino acids based formulas worsen the diarrhea, and the newborns rapidly fail to thrive and develop protein energy malnutrition. Unfortunately, patients have been continued on long-term enteral feeding (EF) without improvement of the diarrhea and with progressive severe protein energy malnutrition. As mentioned above, the same is true for patients treated with immunosuppressive drugs (especially steroids) some time associated with cyclosporine because of mucosal inflammation. Finally, most of the time this neonatal diarrhea, which resists all treatments, requires permanent parenteral nutrition (PN). However, it seems that some infants have a milder phenotype than others [25]. Infants with partial intestinal function and a limited amount of stool output require only partial long-term PN infusions 3–4 times per week. Nevertheless, careful monitoring should be performed to avoid progressive growth retardation.

In most patients, the severity of the intestinal malabsorption and diarrhea make them totally dependent on daily long-term PN, with a subsequent risk of complications. IDE causes intestinal failure that is clearly irreversible in most patients. Liver disease may develop with subsequent end-stage liver cirrhosis in patients with intestinal failure as a consequence of both underlying digestive disease and unadapted PN. Management of patients with intestinal failure requires an early recognition of the condition and analysis of the risk of irreversibility. Thus, in some cases IED is an indication for intestinal transplantation [26-29] and timing of referral for intestinal transplantation is crucial.

The criteria for transplantation have been published in the position paper of the American Society of Transplantation [30] and continue to be debated, especially regarding vascular thrombosis and sepsis. They are more guidelines than formal recommendations and must be balanced with the risks of intestinal transplantation. For instance, in our practice only repeated life-threatening sepsis, especially when complicated with extensive thrombosis, may be a criterion for transplantation. The poor quality of life might serve as indication for intestinal transplantation (ITx), although usual criteria including progressive liver disease, the loss of vascular access, and recurring life-threatening sepsis have not developed. In any case, parents must be extensively informed about the risks of the procedure and about the reasons of any decision [31].

Patients with irreversible intestinal failure and end-stage liver disease (liver cirrhosis) are candidates for a life-saving procedure such as combined liver-small bowel transplantation (LITx). Patients with severe hepatic fibrosis are more difficult to manage [31]. Repeated liver biopsies within 6 to 12 months and careful assessment for portal hypertension are necessary. In addition, it is difficult to assess the amount of functional liver tissue necessary to withstand the insult of portal diversion during the transplantation procedure. Children with severe advanced and progressive hepatic fibrosis are usually listed for LITx. However, some PN-dependent patients with advanced liver dysfunction may experience functional and biochemical liver recovery.

In any case, when long-term PN is effective and well tolerated, it can be used for a prolonged period of time without intestinal transplantation. The long-term prognosis is variable. In general, management should be based on a multidisciplinary approach in centers involving pediatric gastroenterology, parenteral nutrition expertise, home parenteral nutrition program, and liver intestinal transplantation program.

## References

[B1] Cuenod B, Brousse N, Goulet O, De Potter S, Mougenot JF, Ricour C, Guy-Grand D, Cerf-Bensussan N (1990). Classification of intractable diarrhea in infancy using clinical and immunohistological criteria. Gastroenterology.

[B2] Goulet OJ, Brousse N, Canioni D, Walker-Smith JA, Schmitz J, Phillips AD (1998). Syndrome of intractable diarrhoea with persistent villous atrophy in early childhood: a clinicopathological survey of 47 cases. J Pediatr Gastroenterol Nutr.

[B3] Reifen RM, Cutz E, Griffiths AM, Ngan BY, Sherman PM (1994). Tufting enteropathy: a newly recognized clinicopathological entity associated with refractory diarrhea in infants. J Pediatr Gastroenterol Nutr.

[B4] Goulet O, Kedinger M, Brousse N, Cuenod B, Colomb V, Patey N, de Potter S, Mougenot JF, Canioni D, Cerf-Bensussan N (1995). Intractable diarrhea of infancy with epithelial and basement membrane abnormalities. J Pediatr.

[B5] Patey N, Scoazec JY, Cuenod-Jabri B, Canioni D, Kedinger M, Goulet O, Brousse N (1997). Distribution of cell adhesion molecules in infants with intestinal epithelial dysplasia (tufting enteropathy). Gastroenterology.

[B6] Abely M, Hankard GF, Hugot JP, Cezard JP, Peuchmaur M, Navarro J (1998). Intractable infant diarrhea with epithelial dysplasia associated with polymalformation. J Pediatr Gastroenterol Nutr.

[B7] Krantz M, Jansson M, Rectors S, Ryd W, Kristiansson B (1999). Hereditary intractable diarrhea with choanal atresia. A new familial syndrome. J Pediatr Gastroenterol Nutr.

[B8] Djeddi D, Verkarre V, Talbotec C (2002). Tufting enteropathy and associated disorders [abstract]. J Pediatr Gastroenterol Nutr.

[B9] Hoglund P (2006). SLC26A3 and congenital chloride diarrhoea. Novartis Found Symp.

[B10] Hihnala S, Hoglund P, Lammi L, Kokkonen J, Ormala T, Holmberg C (2006). Long-term clinical outcome in patients with congenital chloride diarrhea. J Pediatr Gastroenterol Nutr.

[B11] Muller T, Wijmenga C, Phillips AD, Janecke A, Houwen RH, Fischer H, Ellemunter H, Fruhwirth M, Offner F, Hofer S, Muller W, Booth IW, Heinz-Erian P (2000). Congenital sodium diarrhea is an autosomal recessive disorder of sodium/proton exchange but unrelated to known candidate genes. Gastroenterology.

[B12] Wright EM, Turk E, Martin MG (2002). Molecular basis for glucose-galactose malabsorption. Cell Biochem Biophys.

[B13] Phillips AD, Schmitz J (1992). Familial microvillous atrophy: a clinicopathological survey of 23 cases. J Pediatr Gastroenterol Nutr.

[B14] Ruemmele FM, Schmitz J, Goulet O (2006). Microvillous inclusion disease (microvillous atrophy). Orphanet J Rare Dis.

[B15] Lachaux A, Bouvier R, Loras-Duclaux I, Chappuis JP, Meneguzzi G, Ortonne JP (1997). α6β4 integrin deficiency. A new aetiology for protracted diarrhoea in infancy [abstract]. J Pediatr Gastroenterol Nutr.

[B16] Hermiston ML, Gordon JI (1995). Inflammatory bowel disease and adenomas in mice expressing a dominant negative N-cadherin. Science.

[B17] Murch S, Graham A, Vermault A, Thomson M, Phillips A, Walker-Smith J (1997). Functionnaly significant secondary inflammation occurs in a primary epithelial enteropathy [abstract]. Pediatr Gastroenterol Nutr.

[B18] Beaulieu JF (1992). Differential expression of the VLA family of integrins along the crypt-villus axis in the human small intestine. J Cell Sci.

[B19] Simon-Assmann P, Duclos B, Orian-Rousseau V, Arnold C, Mathelin C, Engvall E, Kedinger M (1994). Differential expression of laminin isoforms and alpha 6-beta 4 integrin subunits in the developing human and mouse intestine. Dev Dyn.

[B20] Simon-Assmann P, Bouziges F, Vigny M, Kedinger M (1989). Origin and deposition of basement membrane heparan sulfate proteoglycan in the developing intestine. J Cell Biol.

[B21] Simo P, Simon-Assmann P, Bouziges F, Leberquier C, Kedinger M, Ekblom P, Sorokin L (1991). Changes in the expression of laminin during intestinal development. Development.

[B22] Simo P, Bouziges F, Lissitzky JC, Sorokin L, Kedinger M, Simon-Assmann P (1992). Dual and asynchronous deposition of laminin chains at the epithelial-mesenchymal interface in the gut. Gastroenterology.

[B23] Simon-Assmann P, Kedinger M (1993). Heterotypic cellular cooperation in gut morphogenesis and differentiation. Semin Cell Biol.

[B24] Ng AY, Waring P, Ristevski S, Wang C, Wilson T, Pritchard M, Hertzog P, Kola I (2002). Inactivation of the transcription factor Elf3 in mice results in dysmorphogenesis and altered differentiation of intestinal epithelium. Gastroenterology.

[B25] Cameron DJ, Barnes GL (2003). Successful pregnancy outcome in tufting enteropathy. J Pediatr Gastroenterol Nutr.

[B26] Lacaille F, Cuenod B, Colomb V, Jan D, Canioni D, Revillon Y, Ricour C, Goulet O (1998). Combined liver and small bowel transplantation in a child with epithelial dysplasia. J Pediatr Gastroenterol Nutr.

[B27] Paramesh AS, Fishbein T, Tschernia A, Leleiko N, Magid MS, Gondolesi GE, Kaufman SS (2003). Isolated small bowel transplantation for tufting enteropathy. J Pediatr Gastroenterol Nutr.

[B28] Goulet O, Sauvat F, Ruemmele F, Caldari D, Damotte D, Cezard JP, Lacaille F, Canioni D, Hugot JP, Berebi D, Sarnacki S, Colomb V, Jan D, Aigrain Y, Revillon Y (2005). Results of the Paris program: ten years of pediatric intestinal transplantation. Transplant Proc.

[B29] Grant D, Abu-Elmagd K, Reyes J, Tzakis A, Langnas A, Fishbein T, Goulet O, Farmer D, on the behalf of the Intestine Transplant Registry (2005). 2003 report of the Intestine Transplant Registry: a new era has dawned. Ann Surg.

[B30] Kaufman SS, Atkinson JB, Bianchi A, Goulet OJ, Grant D, Langnas AN, McDiarmid SV, Mittal N, Reyes J, Tzakis AG, American Society of Transplantation (2001). Indications for pediatric intestinal transplantation: a position paper of the american society of transplantation. Pediatr Transpl.

[B31] Goulet O, Ruemmele F (2006). Causes and management of intestinal failure. Gastroenterol.

